# Closed-Loop Control of a Neuroprosthetic Hand by Magnetoencephalographic Signals

**DOI:** 10.1371/journal.pone.0131547

**Published:** 2015-07-02

**Authors:** Ryohei Fukuma, Takufumi Yanagisawa, Shiro Yorifuji, Ryu Kato, Hiroshi Yokoi, Masayuki Hirata, Youichi Saitoh, Haruhiko Kishima, Yukiyasu Kamitani, Toshiki Yoshimine

**Affiliations:** 1 Department of Neurosurgery, Osaka University Graduate School of Medicine, Suita, Osaka, Japan; 2 Department of Neuroinformatics, ATR Computational Neuroscience Laboratories, Seika-cho, Kyoto, Japan; 3 Graduate School of Information Science, Nara Institute of Science and Technology, Ikoma, Nara, Japan; 4 Division of Functional Diagnostic Science, Osaka University Graduate School of Medicine, Suita, Osaka, Japan; 5 Division of Systems Research, Yokohama National University, Yokohama, Kanagawa, Japan; 6 Department of Mechanical Engineering and Intelligent Systems, The University of Electro-Communications, Chofu, Tokyo, Japan; 7 Department of Neuromodulation and Neurosurgery, Osaka University Graduate School of Medicine, Suita, Osaka, Japan; 8 Graduate School of Informatics, Kyoto University, Kyoto, Japan; Wadsworth Center, UNITED STATES

## Abstract

**Objective:**

A neuroprosthesis using a brain–machine interface (BMI) is a promising therapeutic option for severely paralyzed patients, but the ability to control it may vary among individual patients and needs to be evaluated before any invasive procedure is undertaken. We have developed a neuroprosthetic hand that can be controlled by magnetoencephalographic (MEG) signals to noninvasively evaluate subjects’ ability to control a neuroprosthesis.

**Method:**

Six nonparalyzed subjects performed grasping or opening movements of their right hand while the slow components of the MEG signals (SMFs) were recorded in an open-loop condition. The SMFs were used to train two decoders to infer the timing and types of movement by support vector machine and Gaussian process regression. The SMFs were also used to calculate estimated slow cortical potentials (eSCPs) to identify the origin of motor information. Finally, using the trained decoders, the subjects controlled a neuroprosthetic hand in a closed-loop condition.

**Results:**

The SMFs in the open-loop condition revealed movement-related cortical field characteristics and successfully inferred the movement type with an accuracy of 75.0 ± 12.9% (mean ± SD). In particular, the eSCPs in the sensorimotor cortex contralateral to the moved hand varied significantly enough among the movement types to be decoded with an accuracy of 76.5 ± 10.6%, which was significantly higher than the accuracy associated with eSCPs in the ipsilateral sensorimotor cortex (58.1 ± 13.7%; *p* = 0.0072, paired two-tailed Student’s *t*-test). Moreover, another decoder using SMFs successfully inferred when the accuracy was the greatest. Combining these two decoders allowed the neuroprosthetic hand to be controlled in a closed-loop condition.

**Conclusions:**

Use of real-time MEG signals was shown to successfully control the neuroprosthetic hand. The developed system may be useful for evaluating movement-related slow cortical potentials of severely paralyzed patients to predict the efficacy of invasive BMI.

## Introduction

Restoration of upper limb function by brain–machine interface (BMI) [1,2] is becoming a therapeutic option for severely paralyzed patients [3,4] to improve their daily life [5]. Even in severely paralyzed patients, information about movement intention and movement types for the affected limbs can be extracted from the sensorimotor cortices through neural decoding techniques using invasively recorded signals, such as spikes and electrocorticograms (ECoGs) [1–3,5–7]. However, the decoding accuracy varies significantly among individual patients with paralysis and deteriorates according to the degree of motor dysfunction [7]. It is necessary to evaluate how effectively the brain signals of patients with severe paralysis can control a prosthesis online before invasive BMI systems are used.

In this study, real-time magnetoencephalographic (MEG) signals were used to evaluate individual cortical activity and ability to control a neuroprosthetic hand. Previous studies revealed that some upper limb movements can be inferred from MEG signals [8–10]. Through a source reconstruction technique, MEG signals can enable estimation of the cortical potential in the sensorimotor cortices precisely enough to reconstruct two-dimensional hand movements [11,12]. Notably, the cortical potential is the feature recorded by the ECoG and includes motor information such as trajectory, timing, and types of movements [13–18]. However, the cortical potentials measured by ECoGs are deteriorated among patients with severe paralysis and have decreased accuracy for classifying movement types [7]. The potential performance of an ECoG-based BMI in the context of altered cortical potentials may be estimated from the performance of a MEG-based BMI to classify movement types. No previous studies have demonstrated control of a neuroprosthetic hand with real-time MEG being used to infer the movement types performed at arbitrary time points.

Here, we developed a novel noninvasive BMI system to control a prosthetic hand. The system used real-time MEG to detect movement onset and to classify the movement type online and was applied in six nonparalyzed subjects performing two types of hand movements: grasping and opening. Through use of neural decoding techniques [19], the slow component of the MEG signals enabled successfully inferring the timing and types of the performed movement. The MEG signals and reconstructed cortical potentials during the movements showed a characteristic spatiotemporal pattern of slow cortical potentials (SCPs) in the sensorimotor cortex contralateral to the moved hand. Furthermore, a prosthetic hand was successfully controlled online to grasp and open at the intended time by using the MEG signals. Therefore, the MEG-controlled real-time neuroprosthetic hand was shown to be useful for evaluating the individual ability of patients to control the online BMI.

## Subjects and Methods

### Subjects

Six healthy nonparalyzed subjects (three women, three men; mean age 25.2 years, range 22–31 years), who were all right-handed, participated in this study. All subjects were informed of the purpose and possible consequences of this study, and written informed consent was obtained. The ethics committee of Osaka University Hospital approved this study (no. 12107).

### Recording Method and Data Collection

**Fig 1:**
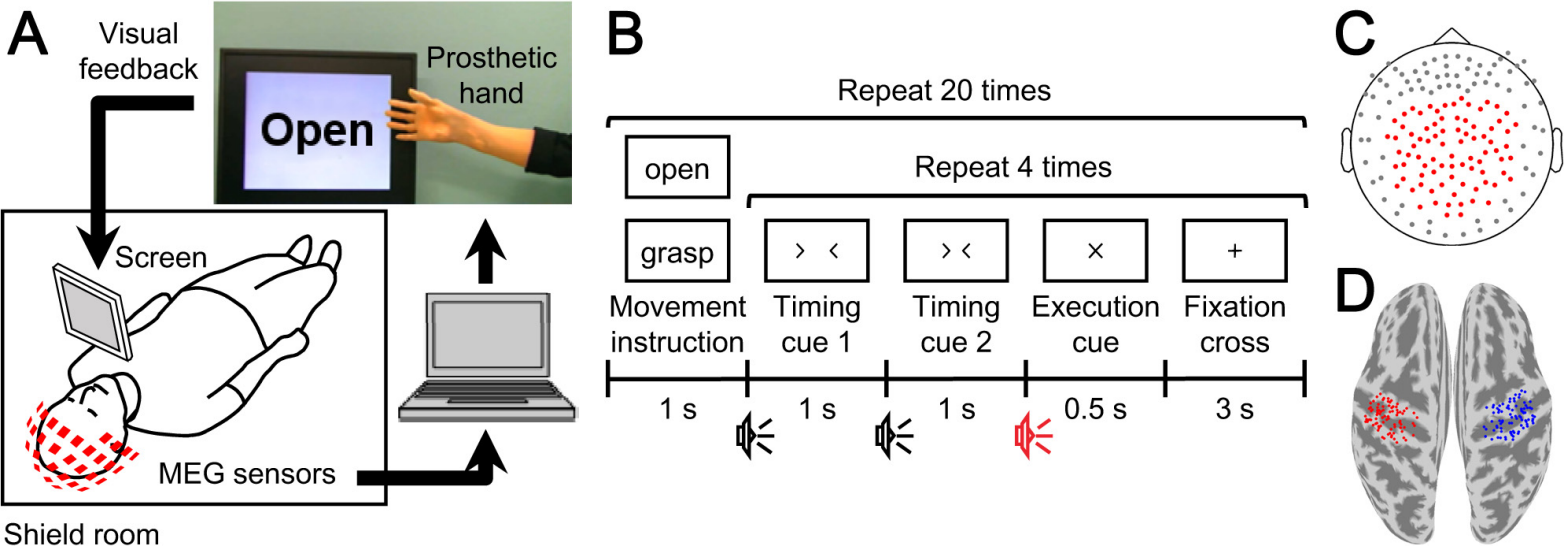
Experiment paradigm and analysis overview. (A) System overview of the real-time prosthetic hand control. MEG signals were acquired in real time to be analyzed on a single computer. The prosthetic hand was controlled according to the decoders that inferred the timing of movement intention and types of performed movements. The subject controlled the prosthetic hand by watching the screen representing the prosthetic hand and following the instructions for movements. (B) Experimental paradigm of the open-loop session. First, one of the movement types, grasping or opening, was presented on the screen in front of the subject. Then the subject moved the right hand as instructed at the execution cue. Each movement type was repeated four times. (C) Position of MEG sensors used for online control and offline analysis are shown as red points. (D) Position of vertices for estimation of eSCPs. Eighty-four vertices were selected in the sensorimotor cortex of each hemisphere: red, contralateral; blue, ipsilateral.

The overall schematic of the system is shown in Fig 1A. Neuromagnetic brain activity was measured by a 160-channel whole-head MEG (MEGvision NEO, Yokogawa Electric Corporation, Kanazawa, Japan) housed in a magnetically shielded room. The subject was in a supine position, and a projection screen was fixed in front of his or her face. A cushion was placed under the elbow of the subject to reduce artifacts caused by shoulder movements. Visual stimuli were shown on the screen by using a visual stimulus presentation system (Presentation, Neurobehavioral Systems, Albany, CA, USA) and a liquid crystal projector (LVP-HC6800, Mitsubishi Electric, Tokyo, Japan). MEG signals were sampled at 1000 Hz with an online low-pass filter at 200 Hz and acquired online by FPGA DAQ boards (PXI-7854R, National Instruments, Austin, TX, USA) after passing through an optical isolation circuit. Subjects were instructed not to move their head to avoid motion artifacts. The head position was measured by five marker coils attached to the subject’s face to estimate cortical current before each session.

### Task

#### Open-loop session

Each subject was given visual and aural execution cues 40 times to grasp or open the right hand once when the cue was given (Fig 1B). To reduce motion artifacts, the subject was instructed to perform the hand movement without moving any other body part. The type of movement to perform was presented visually with either the Japanese word for “grasp” or “open.” After the movement type instruction, four execution cues were given to the subject. The order of the movement type instructions was randomized.

#### Closed-loop session

A screen in front of each subject showed a picture of the prosthetic hand in real time and the instruction monitor as visual feedback (Fig 1A). The instruction monitor displayed either the Japanese word for “grasp” or “open” alternately every 7 s for a total of 22 instructions. Subjects were told to control the prosthetic hand by following the instruction (grasp or open), using the same movements of the right hand as in the open-loop session.

### Experimental Procedure

Each subject was tested in one open-loop session followed by several closed-loop sessions. In the open-loop session, calculation of the decoding features was performed online in order to train an online decoder afterward. For the first few closed-loop sessions, subjects tried to control the prosthetic hand freely. During this period, the experimenter modulated thresholds within the online decoder to detect the onset. Subjects then controlled the prosthetic hand according to the instructions in the closed-loop session with the fixed thresholds (Fig 1A). The performance in controlling the prosthetic hand was evaluated in the last session. The entire experiment required about 1 h to complete.

### Real-Time Decoding and Prosthetic Hand Control

MATLAB R2013a (Mathworks, Natwick, MA, USA) was used for online calculation of decoding features and for online prosthetic hand control. First, MEG signals from 84 parietal sensors (Fig 1C) in the open-loop session were averaged in a 500-ms time window for each sensor and converted into *z*-scores using the means and standard deviations estimated from data recorded during 1000-ms non-overlapping time windows (total 50 windows for 50 s) at the beginning of the open-loop session, when the subjects were instructed to rest. This time-averaged magnetic field, termed the slow magnetic field (SMF), was calculated for the period from −2000 to 1000 ms at 100-ms intervals according to the execution cue.

The SMFs in the open-loop session were used to train the online decoder, which consisted of an onset decoder and class decoder, to control the prosthetic hand online in the subsequent closed-loop session. First, the class decoder was trained by a radial basis function (RBF) kernel support vector machine (SVM) in libsvm toolbox [20] using the SMFs at peak classification accuracy of the movement type estimated by 10-fold cross-validation within ±500 ms of the execution cue. The trained class decoder used the SMF as input in the closed-loop session to provide the inferred movement type, grasp or open. The onset decoder consisted of a mutual information estimator and a movement detector. The mutual information estimator was trained with Gaussian process regression [21] in the GPML toolbox [22] to estimate the mutual information between the actual and inferred movement type using SMFs at three time points from –2000 to 1000 ms: the classification accuracy peak time and time points before and after the peak. These three time points were automatically selected by optimizing the correlation between the mutual information actually obtained in the open-loop session and the mutual information estimated by the trained estimator. Then, to train the movement detector, two time periods, R_off_ and R_on_, were defined in the open-loop data, starting at −2000 ms and the classification accuracy peak time, respectively. The RBF kernel SVM was used to train the detector to classify these two periods. The movement detector received the SMF input at each time point and produced a value that represented the confidence of its being in the R_on_ period. By combining the mutual information and the confidence value with two thresholds, the timing of the movement intention was estimated as the time crossing the threshold. By maximizing the number of correct classifications of movement type at the timing of inferred movement intention within ±500 ms according to the training time of the class decoder, the optimal parameters for the duration of R_off_ and R_on_ periods and the thresholds were determined to train the onset decoder.

In the closed-loop session, the SMF was calculated online every 200 ms to update the prosthetic hand state. Using the latest SMF, the onset decoder estimated the mutual information and confidence value of the movement intention. When both exceeded their respective thresholds, which were manually set before the closed-loop session, movement onset was detected and the prosthetic hand was controlled to make the movement type inferred by the class decoder; otherwise, the prosthetic hand remained still. To avoid multiple onset detections in a single attempt or the execution of a hand movement, the prosthetic hand maintained the inferred movement for 1.5 s after an onset was detected. If no onset was detected for 20 s, the prosthetic hand returned to its resting position.

The prosthetic hand used in this study was developed by Dr. Hiroshi Yokoi to imitate the human upper limb, and each finger has 2 degrees of freedom. The joints are controlled by 10 servo motors using flexible wires. A microcontroller regulated all motors in a coordinated manner to form a grasping or opening hand shape. The overall delay from the MEG system to the visual feedback of the prosthetic hand was around 830 ms: real-time data acquisition, ~20 μs; data processing including the time window for the SMF (500 ms), ~570 ms; prosthetic hand control, ~150 ms; visual feedback, ~110 ms.

### Offline Analysis

#### Classification accuracy in the open-loop session

MEG signals from 84 parietal sensors were converted to SMF by averaging over 500 ms and normalizing to a *z*-score by a 50-s period at the beginning of the session in the same way as the online-acquired features. The cortical potentials estimated by variational Bayesian multimodal encephalography (VBMEG) [23] were likewise converted to the estimated slow cortical potentials (eSCPs) on 84 vertices in the contralateral and ipsilateral sensorimotor cortex (contralateral and ipsilateral eSCPs; Fig 1D). The signal sources for the current estimation were the same 84 sensors used for the SMF, and the sensors-to-vertices conversion was a linear transformation.

Classification accuracy of movement type was estimated by nested cross-validation [24] with SMF or eSCP features calculated for the period from −500 to 500 ms according to the execution cue, with a 500-ms sliding time window, shifted by 100-ms. The nested cross-validation was adopted so that hyperparameters for the SVM and time window to test were always selected independently from the testing data set. To optimize the hyperparameters and the time window, training data sets were classified by 10-fold cross-validation 10 times, and the parameters with the highest averaged classification accuracy of the repeated cross-validation were selected. The classification accuracy was calculated from the classification result of each testing data set, which was tested by the decoder trained with the optimized hyperparameters and time window. All decoding analyses were performed in MATLAB R2007b using RBF kernel SVM.

#### Offline evaluation of onset detection

Ten-fold cross-validation was used to evaluate the accuracy of the onset detection in the open-loop session. The onset decoder was trained by the same algorithm used in the closed-loop session. The timing of the first onset detection was pinpointed in each trial among the test data sets by using the trained onset decoder. The search started at −2000 ms according to the timing at which the class decoder was trained. The SMF and eSCP features were tested at 200-ms intervals until 1000 ms after the timing. Thresholds for the mutual information and confidence value of the movement intention were automatically estimated from the training data set to maximize the number of correctly detected and decoded movements within ±500 ms according to the training time of the class decoder. To evaluate selectivity of the onset detection in the open-loop session, the onset range (−500 to 500 ms, according to the training time of the class decoder) and no-onset range (−2000 to −500 ms) were defined. The selectivity of the onset detection was tested using a one-sided Fisher’s exact test based on detection and no detection during the onset and no-onset ranges.

#### Evaluation of the closed-loop session

The decoding accuracy of movement type in the closed-loop session was defined as the number of prosthetic hand movements that correctly followed the instruction divided by the total number of prosthetic hand movements. To evaluate the selectivity of onset detection in the closed-loop session, the session was divided into two sections depending on the instruction and state of the prosthetic hand: “same state” in which instruction and the state were the same (no need to move the prosthetic hand) and “different state” in which they differed (need to move the prosthetic hand). Selectivity of the onset detection in the closed-loop session was tested using a one-sided Fisher’s exact test based on detection and no detection during the same-state and different-state sections.

## Results

### Movement-Related Activation during the Open-Loop Session

**Fig 2:**
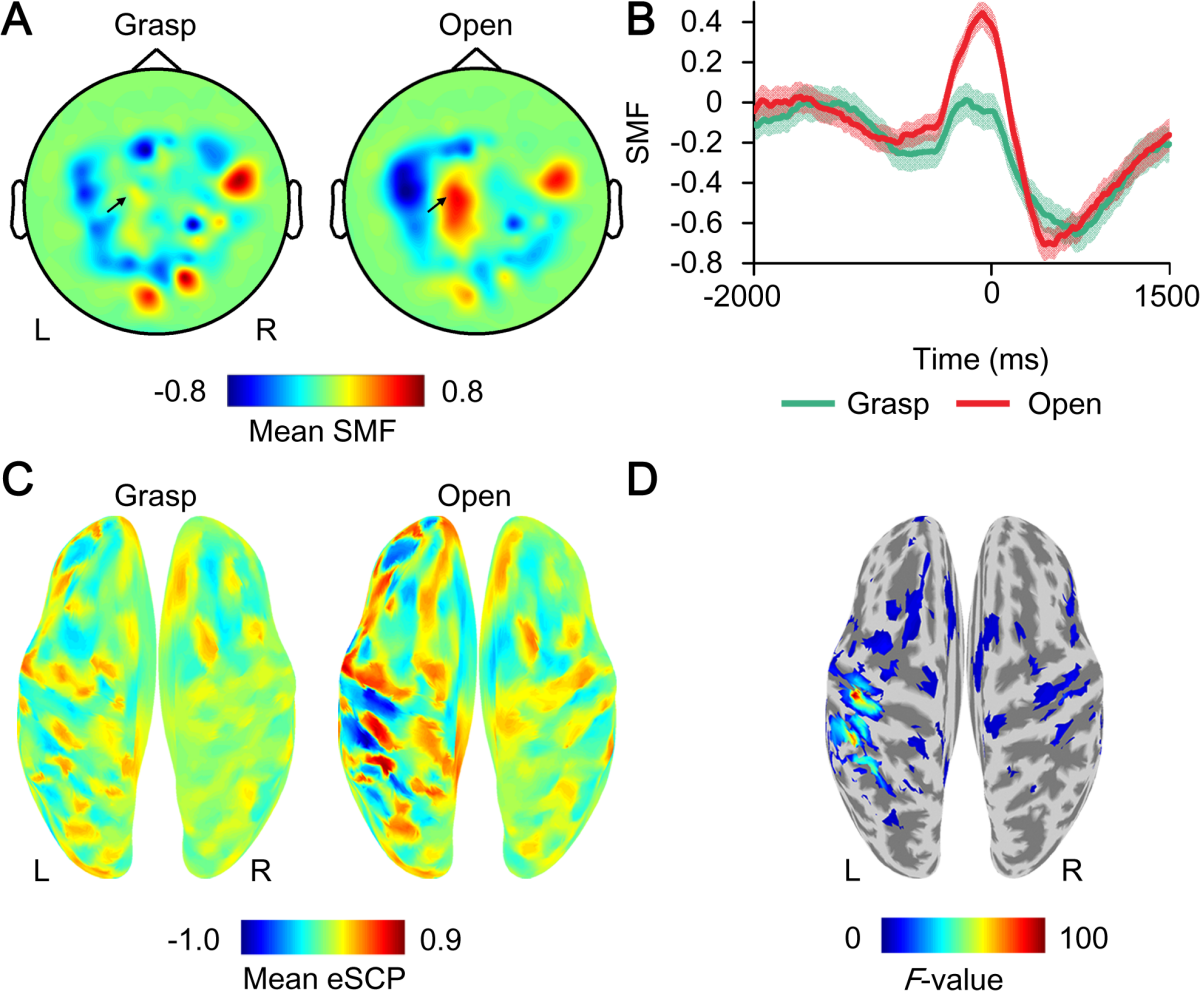
Example of movement-type specific activation during the open-loop session. (A) The *z*-scored MEG signals (SMFs) averaged at the time of execution cue (0 ms) when subject 1 grasped or opened his right hand are color-coded at the location of the sensors. R, right; L, left. (B) The time course of mean SMFs at the sensor indicated by the black arrow in A. The shaded area shows the standard error. (C) The *z*-scored cortical potentials (eSCPs) averaged at 0 ms are color-coded on the normalized brain surface for each movement of subject 1. (D) The *F-*values of one-way ANOVA comparing eSCPs for the two types of movements shown in C are color-coded on the normalized brain surface only for values with *p* < 0.05.

The characteristic activation of MEG signals was observed when subjects moved their hands in the open-loop session. Fig 2A shows a representative mean contour map of the SMF at the time point of execution cues for movement. The map shows the dipole pattern around the sensorimotor cortices. Moreover, the SMF showed a gradual increase from before the execution cue, peaking during movement, and the amplitude of the SMF depended on the movement type (Fig 2B). These spatiotemporal properties of the SMF represent characteristic features of the movement-related cortical field (MRCF) [25] and were similar in all subjects.

To elucidate the signal source of the SMF, the eSCPs were estimated from the same MEG signals using VBMEG. When subject 1 moved his right hand, the eSCP was clearly activated on the left sensorimotor cortex, which was contralateral to the moved hand, depending on the movement type (Fig 2C; for other subjects, see S1 Fig). Differences in the eSCPs between the two types of movements were evaluated by one-way analysis of variance. The *F*-values color-coded on the reconstructed surface of the normalized brain show that the eSCP on the left (contralateral) sensorimotor cortex varied significantly between the movement types (Fig 2D). Notably, a significant *F*-value on the contralateral sensorimotor cortex was observed in all subjects.

### Movement Decoding

**Fig 3:**
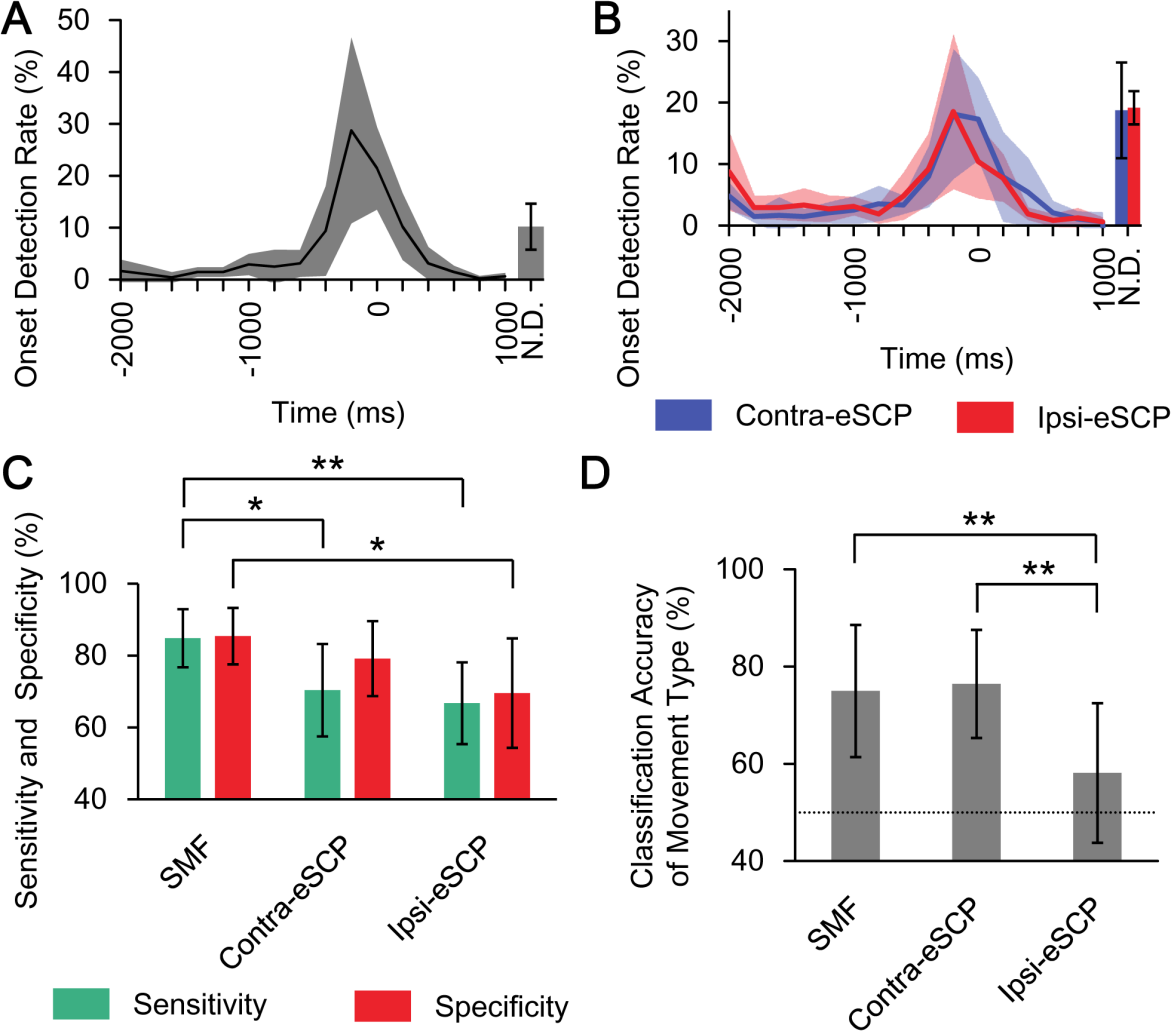
Accuracies for onset detection and classification of movement type. (A) The mean onset detection rate is shown from −2000 to 1000 ms with the standard deviation (*N* = 6). The N.D. (not detected) denotes the rate of trials in which no onset was detected from −2000 to 1000 ms (mean and standard deviation). Time 0 ms denotes target time to detect, which is the training time of the class decoder in training data sets. (B) Blue and red lines show the mean onset detection rates of contra-eSCP and ipsi-eSCP, respectively. The shaded area denotes the standard deviation (*N* = 6). (C) The green and red bars denote the average of the sensitivity and specificity of onset detection, respectively, and the error bars denote 95% confidence intervals. Asterisks show statistical significances (*p < 0.05, **p < 0.01, paired two-tailed Student’s *t*-test, *N* = 6) (D) The classification accuracies of movement type were compared among three types of features for decoding (**p < 0.01, paired two-tailed Student’s *t*-test, *N* = 6). The mean and 95% confidence interval are shown. Dotted line denotes chance level (50%).

The timing of movement intention was inferred by the onset decoder using the SMFs for each trial in the open-loop session (−2000 to 1000 ms) (see Subjects and Methods). To evaluate the accuracy of onset detection, the earliest timing of the onset detection in each trial was evaluated by 10-fold cross-validation. Fig 3A demonstrates that the timing of movement intention was selectively inferred within ±500 ms and peaked at –200 ms, using SMFs (time 0 ms corresponds to the timing at which the class decoder was trained using the SMF). 72.9 ± 12.9% (mean ± SD) of the onset was selectively detected within ±500 ms, with statistical significance for all subjects (*p* < 0.05, one-sided Fisher’s exact test; see also S1 Table). Notably, 56.7 ± 12.9% and 47.7 ± 17.6% of the onsets were selectively detected within ± 500 ms using the eSCP of the sensorimotor cortex contralateral (contra-eSCP) and ipsilateral (ipsi-eSCP) to the tested right hand, respectively, with statistical significance for five out of the six subjects (*p*<0.05, one-sided Fisher’s exact test) (Fig 3B). Moreover, the sensitivity and specificity of the detection was the best using the SMF, compared to using contra-eSCP and ipsi-eSCP (Fig 3C). Thus, the onset decoder using the SMF succeeded in inferring the time point at which the class decoder infers the type of performed movement.

The class decoder inferred the performed movement types in the open-loop session (see Subjects and Methods). The movement type was classified by using the SMFs with accuracies of 75.0 ± 12.9% (mean ± SD), which significantly exceeded accuracy by chance (*p* < 0.05, one-sided binominal test) in five of the six subjects (Fig 3D, see also S1 Table). Moreover, to evaluate the origin of the motor information, the classification accuracies were evaluated using contra-eSCP and ipsi-eSCP. The classification accuracies using contra-eSCPs were 76.5 ± 10.6%, which were comparable to those using the SMFs, and were significantly superior to those using the ipsi-eSCPs (58.1 ± 83.7%; *p* = 0.0072, paired two-tailed Student’s *t-*test; Fig 3D, see also S2 Table). These results suggest that the motor information for classifying the movement types mostly originated in the sensorimotor cortex contralateral to the tested hand.

### Online Control of the Prosthetic Hand

**Fig 4:**
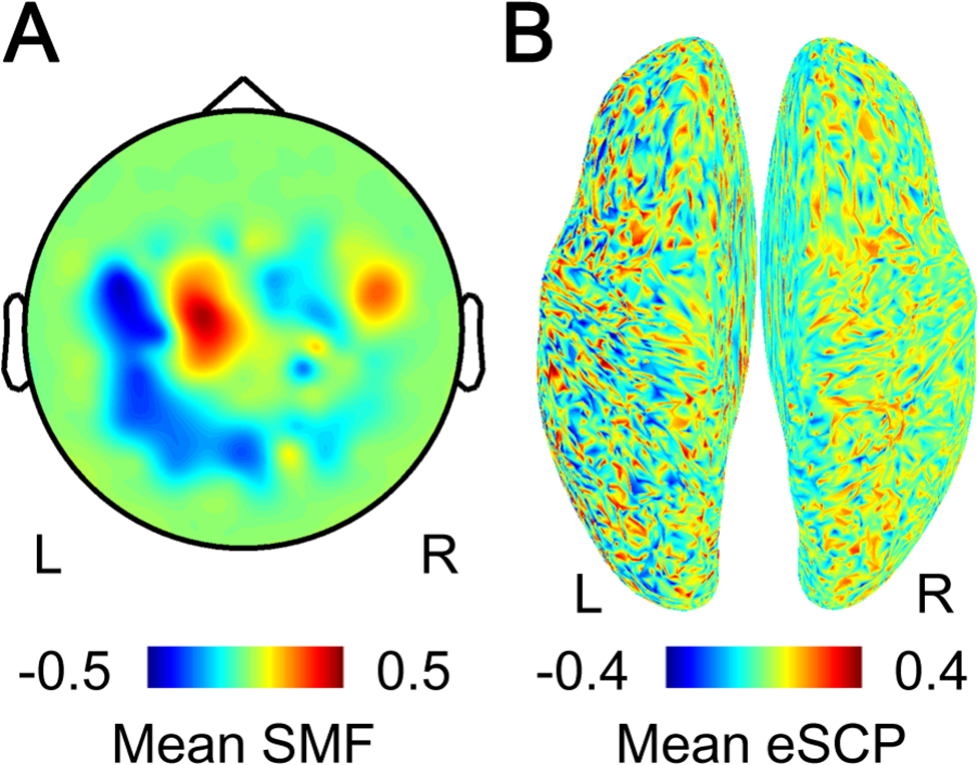
SMF and eSCP at the timing of detected onset. (A) The SMF when movement onset was detected is color-coded at the location of the sensors. R, right; L, left. (B) The eSCP at the timing of the detection is color-coded on the normalized brain surface.

Using the onset detection and movement type classification in real time, the prosthetic hand could be controlled by the SMFs in the closed-loop condition. For example, subject 1 successfully controlled the prosthetic hand in 10 of 12 total movements following the instructions (83.3%; S1 Video). The SMF and eSCP at the timing of the detected onset were estimated offline, by adapting the online decoder to the MEG signals during the closed-loop session. The SMF exhibited a dipole pattern (Fig 4A), and the eSCP showed activation of the sensorimotor cortex contralateral to the tested right hand (Fig 4B). For all subjects, the prosthetic hand was successfully moved according to instructions in more than 50% of the prosthetic hand movements with a few onset detections of 1.21 ± 0.45 (mean ± SD) times on average. Moreover, the movement onset was selectively detected during the specific periods, in which the subject was instructed to initiate movements, with a statistical significance for 50% of subjects (*p* < 0.05, one-sided Fisher’s exact test). The accuracy of classifying the movement type also significantly exceeded chance for four of the six subjects (*p* < 0.05, one-sided binominal test; Table 1). These results demonstrated that the neuroprosthetic hand was successfully controlled using the SMFs in the closed-loop condition.

**Table 1:** Summary of closed-loop prosthetic hand control.

Subject	Detection of movement onset		Decoding of movement type
	Sensitivity (%)	Specificity (%)	Accuracy (%)
1	88.2	64.7	83.3*
2	85.0	40.0	72.2*
3	66. 7	70.6	76.9*
4	64.0	50.0	52.4
5	52.6	60.0	60.0
6	77.8	66. 7	78.6*

*p < 0.05 as compared to chance (50%).

## Discussion

In this study, we developed a noninvasive BMI to control a neuroprosthetic hand using SMFs recorded from real-time MEG. The SMFs had the characteristics of MRCFs and were successfully decoded to infer the timing and type of the performed movement. Notably, the eSCPs estimated from the SMFs showed movement type–specific activation in the contralateral sensorimotor cortex to the moved hand, with the decoding accuracy for inferring movement type being significantly superior to that in the ipsilateral sensorimotor cortex; thus, the motor information of the SMFs was estimated to originate mainly from the SCPs in the contralateral sensorimotor cortex. Finally, real-time decoding using the SMFs was successfully used to control the neuroprosthetic hand. The BMI using SMFs developed in this study may be used to noninvasively evaluate the potential efficacy of invasive BMI using the SCPs of the paralyzed patients.

### Characteristics of Our Proposed BMI

Compared to other noninvasive BMIs previously reported, our proposed BMI is characterized by the use of two types of motor information to control the prosthetic hand: movement intention and movement type. Although previous studies have shown that movement types can be decoded by noninvasive measurements such as functional magnetic resonance imaging [26], functional near-infrared spectroscopy [27], electroencephalogram [10,28], and MEG [8–11,28], the decoding to infer the movement types has not been used to control the neuroprosthetic hand online. However, decoding to infer movement intention or timing to move has frequently been used in other studies to control various external devices [29–34]. Combining these two types of motor information, our proposed BMI allows users to control a prosthetic hand simply by performing the intended movements at the desired times.

Moreover, the proposed BMI will be suitable for adapting BMI control to individual abilities. Our previous study using ECoG demonstrated that decoding accuracies to infer the movement types were deteriorated among severely paralyzed patients compared to nonparalyzed subjects, but the accuracies to infer the movement intention were preserved even in severely paralyzed patients [7]. Moreover, in many previous studies with noninvasive BMI, the efficacy of using movement intention to control a prosthesis [30,31] was successfully shown for patients with motor dysfunction [32,33]. These studies showed that the information inferring the movement intention was necessary for a clinically practical BMI, and that it was preserved in paralyzed patients. A BMI that can infer the movement type will enable more sophisticated control of a prosthesis, including two-dimensional estimation of fingertip movements [11]; however, the application of the system will be restricted to patients whose brain signals are suitable for neural decoding. Therefore, by combining information for movement intention and movement type, our proposed BMI can be adapted for individual patients based on how much motor information can be derived from their brain signals.

Notably, the accuracies for classifying the two types of movements were relatively low compared to our previous reports [9]. Though, some recent reports have pointed out the possibility of over-estimation of accuracy without using nested cross-validation [35,36], it is worth noting that our method for classifying movements successfully exceeded the chance level, even using strict evaluations. On the other hand, in the case of the closed-loop control, the classification accuracy might be affected by some limitations of our system. The system has a delay of approximately 830 ms to control the prosthetic hand by an intention. Moreover, the MEG signals were evaluated only every 200 ms, which is rather slow compared to the variances of the cortical signals. The classification accuracies online might be increased by improving the speed of the system.

In addition, the performance and the applicability of noninvasive BMIs could be improved by advances in recording technology and the decoding methods. Recent studies demonstrated that optically pumped magnetometers will became an alternative to the superconducting quantum interference devices that require cryogenic cooling and prevent the development of a portable MEG-BMI suitable for daily use [37,38]. Moreover, a recent EEG study demonstrated that an amputee successfully controlled a neuroprosthesis in real-time using slow components of the signals [39]. These advances may enable noninvasive BMIs to restore the motor functions for severely paralyzed patients.

### Preoperative Evaluation Using MEG-based BMI

The performance of our MEG-based neuroprosthesis might reflect a patient’s ability to control a BMI using the SCPs in the sensorimotor cortex. The source localization analysis showed that the SMFs for controlling the neuroprosthetic hand had characteristics of the MRCF and corresponded to the SCPs in the sensorimotor cortex contralateral to the moved hands. Moreover, the decoding analysis suggested that the motor information of the SMFs largely originated from the SCPs in the contralateral sensorimotor cortex. Therefore, using the signals and motor information from a common origin, our MEG-based BMI may enable estimating the ability of severely paralyzed patients to control an ECoG-based BMI using the SCPs in the contralateral sensorimotor cortex, although further studies are necessary to elucidate their relations.

## Conclusions

The proposed BMI demonstrated real-time noninvasive control of a prosthetic hand, using motor information about movement intention and movement type. This BMI system might permit preoperative evaluation of invasive BMIs using the SCPs, which is essential for the clinical application of the BMI.

## Supporting Information

S1 Fig Movement-type specific activation during the open-loop session. SMFs, eSCPs, and *F*-values at the timing of execution cue are shown for subjects 2 to 6.
(PDF)

S1 Table Summary of movement type classification and movement onset detection results using SMF. (PDF)

S2 Table Summary of accuracy to classify movement type using eSCP. (PDF)

S3 Table Details of closed-loop prosthetic hand control. (PDF)

S1 Video Real-time control of a prosthetic hand. Video shows the subject 1 controlling a prosthetic hand by moving his right hand to follow instructions displayed onto the monitor.
(MP4)
