# Supplementary material for: Closed-Loop Control of a Neuroprosthetic Hand by Magnetoencephalographic Signals
Source: PLoS One. 2015 Jul 2;10(7):e0131547. doi: 10.1371/journal.pone.0131547 (PMC4489903; doi:10.1371/journal.pone.0131547)
Supplement: S1 Fig — SMFs, eSCPs, and F-values at the timing of execution cue are shown for subjects 2 to 6. (PDF) [file pone.0131547.s001.pdf]

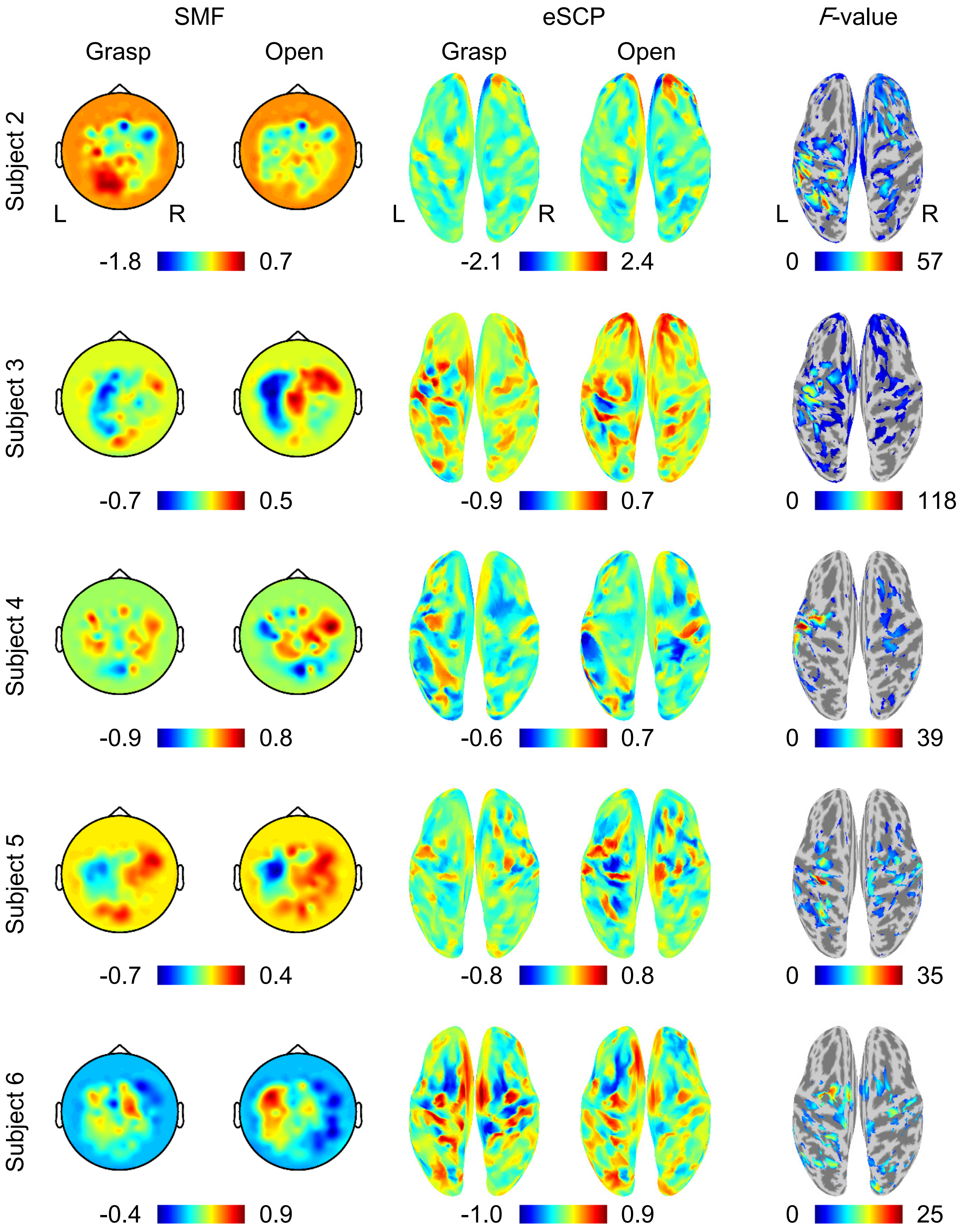

S1 Fig. Movement-type specific activation during the open-loop session.
SMF, eSCP, and *F*-value at the timing of execution cue are shown for subjects 2 to 6.
