# Supplementary material for: Closed-Loop Control of a Neuroprosthetic Hand by Magnetoencephalographic Signals
Source: PLoS One. 2015 Jul 2;10(7):e0131547. doi: 10.1371/journal.pone.0131547 (PMC4489903; doi:10.1371/journal.pone.0131547)
Supplement: S1 Table — (PDF) [file pone.0131547.s002.pdf]

S1 Table. Summary of movement type classification and movement onset detection results using SMF.

Subject	Classification of movement type	Detection of movement onset		
	Accuracy (%)	Sensitivity (%)	Specificity (%)	p-value
1	86.3**	89.0	91.3	< 0.001
2	90.0**	97.4	96.3	< 0.001
3	82.5**	84.6	81.3	< 0.001
4	58.8	75.0	75.0	< 0.001
5	63.8**	82.1	83.8	< 0.001
6	68.8**	80.9	85.0	< 0.001

**$p < 0.01$ as compared to chance (50%); p-value: p-value of one-sided Fisher's exact test.
