# Supplementary material for: Closed-Loop Control of a Neuroprosthetic Hand by Magnetoencephalographic Signals
Source: PLoS One. 2015 Jul 2;10(7):e0131547. doi: 10.1371/journal.pone.0131547 (PMC4489903; doi:10.1371/journal.pone.0131547)
Supplement: S2 Table — (PDF) [file pone.0131547.s003.pdf]

S2 Table. Summary of accuracy to classify movement type using eSCP.

Subject	Classification accuracy of movement type (%)	
	Contralateral eSCP	Ipsilateral eSCP
1	87.5**	57.5
2	82.5**	72.5**
3	87.5**	71.3**
4	68.8**	37.5
5	68.8**	62.5*
6	63.8**	47.5

$^{*}p < 0.05$, $^{**}p < 0.01$ as compared to chance (50%).
