# Supplementary material for: Closed-Loop Control of a Neuroprosthetic Hand by Magnetoencephalographic Signals
Source: PLoS One. 2015 Jul 2;10(7):e0131547. doi: 10.1371/journal.pone.0131547 (PMC4489903; doi:10.1371/journal.pone.0131547)
Supplement: S3 Table — (PDF) [file pone.0131547.s004.pdf]

S3 Table. Details of closed-loop prosthetic hand control.

Subject	Decoding of movement type		Detection of movement onset				
	# of correct movements	# of total movements	# of TP	# of FP	# of FN	# of TN	p-value
1	10	12	15	6	2	11	0.002
2	13	18	17	12	3	8	0.078
3	10	13	14	5	7	12	0.024
4	11	21	16	10	9	10	0.261
5	6	10	10	6	9	9	0.350
6	11	14	14	6	4	12	0.009

TP: true positive, TN: true negative, FP: false positive, FN: false negative for onset detection; p-value: p-value of one-sided Fisher's exact test.
